# Incorporação da Litotripsia Intracoronária na Prática Clínica: Uma Análise Comparativa Temporal de Segurança, Eficácia e Curva de Aprendizado

**DOI:** 10.36660/abc.20250783

**Published:** 2026-09-08

**Authors:** Guy F. A. Prado, Stefano Garzon, José Mariani, Willterson Bandeira, Felipe Mateus Teixeira Bezerra, Breno Oliveira Almeida, Gustavo Neves de Araujo, Ana Claudia Conrado, João Silveira, Emanuele Barbato, Flavio Ribichini, Pedro Alves Lemos

**Affiliations:** 1 Hospital Israelita Albert Einstein São Paulo SP Brasil Hospital Israelita Albert Einstein, São Paulo, SP – Brasil; 2 University of Rome La Sapienza Department of Clinical and Molecular Medicine Rome Itália University of Rome La Sapienza - Department of Clinical and Molecular Medicine, Rome – Itália; 3 Universita degli Studi di Verona Scuola di Medicina e Chirurgia Verona Itália Universita degli Studi di Verona Scuola di Medicina e Chirurgia – Cardiology, Verona – Itália; 4 Unimed Grande Florianópolis Florianópolis SC Brasil Unimed Grande Florianópolis, Florianópolis, SC – Brasil; 5 Centro Hospitalar Universitario do Porto Porto Portugal Centro Hospitalar Universitario do Porto EPE, Porto – Portugal

**Keywords:** Litotripsia, Curva de Aprendizado, Ultrassonografia de Intervenção, Intervenção Coronária Percutânea

## Abstract

**Fundamento:**

A litotripsia intravascular (IVL) surgiu como uma tecnologia inovadora para o tratamento de lesões coronárias calcificadas.

**Objetivo:**

Avaliar se a seleção de casos e o desempenho intervencionista com a IVL evoluíram ao longo do tempo após sua incorporação à prática clínica.

**Métodos:**

Incluímos prospectivamente 104 pacientes consecutivos com 127 lesões coronárias calcificadas tratadas por meio da IVL. A coorte foi dividida em dois grupos temporais (52 pacientes cada), com base na ordem de inclusão dos pacientes. Dados clínicos e o desempenho do procedimento foram comparados entre os grupos. Todos os testes estatísticos foram bicaudais, com nível de significância de 0,05.

**Resultados:**

A idade média foi de 73,2 ± 10,2 anos e 62,5% dos pacientes apresentavam síndromes coronárias agudas. Foram identificadas calcificação grave e lesões do tipo B2/C em 73,2% e 98,4% dos casos, respectivamente. A IVL foi realizada como estratégia inicial em 92,9% das lesões; utilizou-se imagem intravascular em 78% dos casos e aterectomia rotacional em 6,3%, sendo obtido sucesso angiográfico em 96,0%. Em comparação com a primeira coorte, a segunda apresentou mais lesões em bifurcação (60,0% vs. 41,9%; p = 0,04), mais intervenções na artéria circunflexa (24,6% vs. 8,1%; p = 0,02) e menor uso de balões com 4,0 mm de diâmetro (4,6% vs. 18,5%; p = 0,02). A segunda coorte abrangeu um período de inclusão mais longo (168 ± 90,8 vs. 120 ± 72,8 dias; p = 0,004) e apresentou menos complicações angiográficas (1,5% vs. 14,5%; p = 0,01), enquanto a incidência de eventos cardíacos adversos maiores intra-hospitalares não diferiu (3,8% vs. 5,8%; p = 1,0).

**Conclusão:**

A IVL é uma modalidade eficaz para a modificação do cálcio coronário. As mudanças temporais na seleção de casos e no desempenho intervencionista podem refletir uma curva de aprendizado e adaptação.

## Graphical abstract

**Figura Central:**
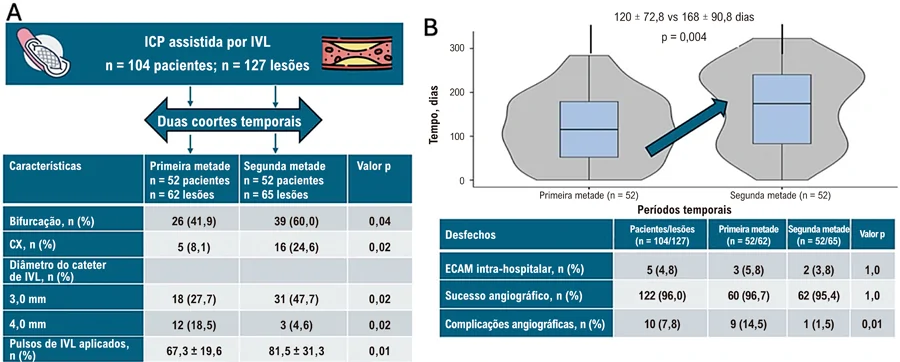
Incorporação da Litotripsia Intracoronária na Prática Clínica: Uma Análise Comparativa Temporal de Segurança, Eficácia e Curva de Aprendizado

Incorporação da Litotripsia Intracoronária à Prática Clínica. Experiência inicial com ICP assistida por IVL para o tratamento de lesões coronárias calcificadas. (A) As duas coortes temporais de pacientes consecutivos apresentaram diferenças significativas nas características angiográficas e procedimentais. (B) O período inicial apresentou maior incidência de complicações angiográficas, embora não tenham sido observadas diferenças quanto a ECAM. A segunda metade apresentou um período de inclusão de pacientes mais longo, refletindo um uso mais seletivo da terapia com IVL. Observaram-se mudanças na seleção de casos e no desempenho intervencionista ao longo da adoção da IVL. CX: artéria circunflexa esquerda; ECAM: eventos cardíacos adversos maiores; ICP: intervenção coronária percutânea; IVL: litotripsia intravascular.

## Introdução

A prevalência de lesões coronárias calcificadas em pacientes submetidos à intervenção coronária percutânea (ICP) tem aumentado progressivamente devido à idade avançada e à presença de múltiplas comorbidades.^1^ A calcificação coronária grave representa um desafio significativo para a ICP, pois restringe o cruzamento e a entrega de dispositivos intracoronários, prolonga o tempo do procedimento, prejudica a expansão ideal do stent e aumenta o risco de dissecção ou ruptura do vaso. Além disso, a manipulação de stents coronários através de segmentos calcificados pode danificar o revestimento polimérico e a plataforma metálica, bem como o sistema de entrega. Em suma, a presença de calcificação grave acarreta um risco aumentado não apenas de complicações agudas, mas também de piores desfechos clínicos a longo prazo.^2^

Diversos dispositivos e técnicas foram desenvolvidos para facilitar a ICP de lesões calcificadas, incluindo balões não complacentes e balões de escore (*scoring balloons*), aterectomia rotacional e orbital e terapia com laser *excimer*. A escolha da estratégia terapêutica ideal é geralmente orientada por um fluxo de trabalho estruturado e baseado em consensos para o manejo atual de lesões coronárias calcificadas.^3^ No entanto, ainda existem várias limitações, tornando esse subgrupo específico de lesões um dos mais desafiadores, frequentemente exigindo alto nível de experiência dos operadores.

Foi desenvolvida a litotripsia intravascular (IVL) como uma ferramenta dedicada ao tratamento dos vasos calcificados. A IVL incorpora as mesmas propriedades físicas da litotripsia extracorpórea para nefrolitíase, utilizando energia elétrica para gerar ondas sonoras que fraturam seletivamente o cálcio superficial e profundo na parede do vaso, por meio de um balão semicomplacente em baixa pressão (4 a 6 atmosferas).^4^ Essa tecnologia foi avaliada em múltiplos estudos e incorporada progressivamente à prática clínica após aprovação regulatória em diferentes jurisdições, com base em ensaios prospectivos e registros do mundo real.^5,6^

O desempenho de tecnologias intervencionistas pode diferir daquele observado em ensaios clínicos quando aplicado à prática de rotina, particularmente em cenários de alta complexidade, tais como lesões coronárias gravemente calcificadas. Ademais, toda nova tecnologia na área médica pode envolver uma curva de aprendizado. Esse contexto motivou o presente estudo, que visou avaliar a experiência inicial em vida real com a ICP assistida por IVL após a incorporação dessa tecnologia à prática diária em um centro cardiovascular de alto volume. Especificamente, buscamos avaliar a segurança e a eficácia da IVL em pacientes consecutivos com lesões coronárias calcificadas e investigar se a seleção de casos e o desempenho do procedimento mudaram ao longo do tempo, como um indicador de uma possível curva de aprendizado e adaptação.

## Métodos

### Pacientes e desenho do estudo

Após sua aprovação no Brasil em junho de 2022, a IVL foi incorporada ao arsenal intervencionista do nosso centro. O presente estudo inclui todos os pacientes consecutivos tratados com a nova tecnologia na instituição, desde o caso inicial (que foi o primeiro realizado no Brasil; Figura 1) até janeiro de 2024. Todos os pacientes admitidos com síndrome coronariana crônica ou aguda que receberam tratamento com IVL para lesões coronarianas gravemente calcificadas foram incluídos no estudo como parte de um registro prospectivo de centro único. Adicionalmente, para avaliar a proficiência dos operadores, a coorte foi dividida em dois grupos de tamanho semelhante com base na ordem temporal de inclusão dos pacientes (primeira e segunda metades). Os grupos foram comparados quanto às características clínicas e do procedimento, bem como à ocorrência de desfechos adversos. A decisão de realizar a ICP ficou a critério dos operadores, cardiologistas clínicos ou da *heart team* institucional, seguindo as diretrizes internacionais vigentes para a revascularização adequada. A indicação da IVL, seja como estratégia inicial ou de resgate — por exemplo, após falha da dilatação com balão não complacente de alta pressão — bem como sua combinação com outros dispositivos de modificação do cálcio (como aterectomia rotacional ou balões de corte), seguiu, em geral, as recomendações baseadas em consensos atuais para o manejo de lesões coronarianas gravemente calcificadas.^3^ No entanto, dado o desenho pragmático do presente registro prospectivo e a ausência de um protocolo de estudo específico, a decisão final de utilizar a IVL coube aos operadores, refletindo a prática diária em um laboratório de hemodinâmica de alto volume.

**Figura 1:**
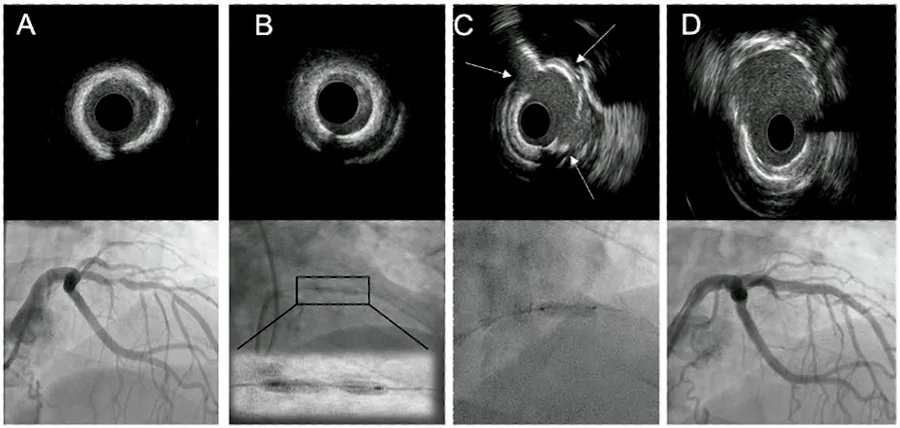
– O primeiro paciente tratado no Brasil, em 13 de junho de 2022, com intervenção coronária percutânea assistida por litotripsia intravascular, em uma estratégia de resgate bem-sucedida. O painel superior em (A) mostra a imagem de IVUS de um arco de cálcio na DAE, e o painel inferior em (A) apresenta a angiografia de uma lesão calcificada crítica na DAE. Em (B), o painel superior mostra a imagem de IVUS ilustrando a persistência do cálcio após a dilatação com balão não complacente em alta pressão na DAE, e o painel inferior apresenta a angiografia de uma dilatação incompleta com balão não complacente em alta pressão na mesma lesão. Em (C), o painel superior mostra marcas brancas estreitas indicando a fratura do cálcio identificada pelo IVUS na DAE, e o painel inferior exibe a angiografia do balão de litotripsia (3,5 × 12 mm) emitindo 80 (8 × 10) pulsos de ondas acústicas na mesma lesão. Por fim, em (D), o painel superior revela o resultado final após o implante de dois stents farmacológicos, avaliado por IVUS, enquanto o painel inferior mostra o resultado angiográfico final. A ALM da DAE antes e após o procedimento foi de 2,36 mm2 e 11,32 mm2, respectivamente. ALM: área luminal mínima; DAE: artéria descendente anterior esquerda; IVUS: ultrassom intravascular.

O presente estudo seguiu os princípios da Declaração de Helsinque e obteve aprovação do comitê de revisão e do comitê de ética do Hospital Israelita Albert Einstein. Todos os pacientes foram contatados para obtenção do consentimento informado, pessoalmente ou por e-mail; para aqueles que não responderam, o comitê de ética local dispensou a exigência de consentimento informado quanto à coleta, análise e publicação da presente análise de estudo não intervencional.

### Sistema de litotripsia intravascular coronária

O cateter de IVL coronariana (Shockwave Medical, Santa Clara, Califórnia, EUA) é um cateter de troca rápida que contém dois emissores de litotripsia integrados a um balão coronário semicomplacente (disponível em diâmetros de 2,5 a 4,0 mm e comprimento de 12 mm). Durante o procedimento, o balão de IVL preenchido com contraste diluído é inflado no segmento-alvo (4 atmosferas), seguido da aplicação local de ondas de pressão sônica pelos emissores, que criam um efeito de campo circunferencial visando fraturar seletivamente o cálcio e alterar a complacência do vaso. O gerador é pré-programado para liberar rajadas de 10 pulsos (1 pulso/s), até um máximo de 80 pulsos por cateter.^7^ Para a presente série inicial de pacientes, o novo cateter de IVL C2 plus, com capacidade para 120 pulsos, estava indisponível.

### Intervenção coronária percutânea

Todos os procedimentos foram realizados exclusivamente por cinco operadores experientes em ICP complexa. Os pacientes receberam pelo menos 100 mg de ácido acetilsalicílico antes do procedimento. Para aqueles que não estavam em uso de terapia antiplaquetária dupla, administrou-se uma dose de ataque — preferencialmente de prasugrel ou ticagrelor, ou, alternativamente, de clopidogrel — após a ICP. Os pacientes foram orientados a manter a terapia antiplaquetária dupla por um período mínimo de 6 meses em casos de doença coronária estável e de 1 ano em casos de síndromes coronárias agudas, em conformidade com as diretrizes vigentes.^8^ Dada a natureza complexa dos procedimentos, incentivou-se fortemente o uso de ultrassom intravascular (IVUS) ou tomografia de coerência óptica (OCT) para guiar a ICP; no entanto, a utilização de métodos de imagem ficou a critério do operador.

### Desfechos e definições

Para avaliar o desempenho clínico e procedimental, os pacientes foram monitorados quanto ao desfecho primário de eventos cardíacos adversos maiores (ECAM) intra-hospitalares, definido como o composto de morte cardíaca, infarto do miocárdio e revascularização do vaso-alvo. O estudo também coletou dados sobre complicações angiográficas ou relacionadas ao dispositivo, incluindo perfuração do vaso, oclusão aguda do vaso, oclusão aguda de ramo lateral, *no-reflow*, fluxo lento (*slow flow*), aprisionamento do balão de IVL, ruptura do balão de IVL, dissecções coronárias e fibrilação ventricular. Complicações coronárias graves foram definidas como: (i) dissecções tipo D, E ou F; (ii) oclusão do vaso; (iii) qualquer perfuração coronária; e (iv) fluxo lento/*no-reflow*. O sucesso angiográfico foi definido como estenose residual final após ICP < 30% pela análise coronária quantitativa (QCA), em combinação com fluxo grau 3 na classificação Thrombolysis in Myocardial Infarction (TIMI) e ausência de complicações angiográficas graves, adaptado a partir de definições de consensos anteriores.^9^

O infarto do miocárdio foi definido de acordo com a Quarta Definição Universal de Infarto do Miocárdio.^10^ A trombose de stent foi definida em conformidade com as diretrizes do Academic Research Consortium.^11^ Adotou-se a classificação de morfologia da lesão do American College of Cardiology/American Heart Association.^12^

A calcificação angiográfica grave foi definida como radio-opacidades observadas sem movimento cardíaco, afetando tipicamente ambos os lados do lúmen arterial. A calcificação grave avaliada por IVUS ou OCT foi definida como calcificação excedendo 270° ou 180° dos contornos do vaso, respectivamente, com extensão de pelo menos 5 mm ou espessura de cálcio visualizada por OCT superior a 500 μm.

### Análise estatística

As variáveis categóricas são apresentadas como contagens (porcentagens). As variáveis contínuas são apresentadas como média ± desvio-padrão, quando apresentavam distribuição normal, e como mediana (intervalo interquartil), nos demais casos. A normalidade foi avaliada utilizando o teste de Shapiro–Wilk e a inspeção visual de histogramas e gráficos Q–Q. As comparações entre grupos para variáveis categóricas foram realizadas utilizando o teste do qui-quadrado ou o teste exato de Fisher, conforme apropriado. As variáveis contínuas foram comparadas entre grupos independentes utilizando o teste t de Student não pareado para dados com distribuição aproximadamente normal e o teste U de Mann–Whitney para dados sem distribuição normal. Como os desfechos se limitaram a eventos ocorridos durante a hospitalização índice, não foram realizadas análises de tempo até o evento (Kaplan–Meier); as taxas de eventos foram comparadas entre as coortes temporais utilizando o teste exato de Fisher. Não foram realizados cálculos de tamanho amostral, uma vez que o estudo derivou de um registro de centro único. Todas as análises foram realizadas utilizando o software Jamovi^®^ (versão 2.5.7; The Jamovi Project, Sydney, Austrália). Todos os testes de hipótese foram bicaudais, com nível de significância de 0,05.

## Resultados

No total, foram incluídos 104 pacientes com 127 lesões. A idade média foi de 73,2 ± 10,2 anos; 76% eram do sexo masculino, 62,5% apresentavam síndrome coronariana aguda e 44,2% tinham diabetes (Tabela 1). Observou-se doença arterial coronariana multivascular em 77,9% dos pacientes, enquanto lesões do tipo B2/C e calcificação grave à fluoroscopia foram identificadas em 73,2% e 98,4% dos casos, respectivamente (Tabela 2). O comprimento médio da lesão foi de 21,7 ± 11,7 mm, com envolvimento de bifurcação em 51,1% e doença de tronco da coronária esquerda não protegido em 7,1% das lesões (Tabela 2). A IVL foi utilizada como estratégia de tratamento inicial em 92,9% das lesões, sendo as demais tratadas como estratégia de resgate após pré-dilatação incompleta com balão. Foi utilizada imagem intravascular em 78,0% dos casos. A aterectomia rotacional foi realizada em 8 (6,3%) lesões antes da terapia com IVL, como tratamento adjuvante para facilitar a passagem do balão de IVL. Balões de corte foram utilizados em 29 lesões (22,8%), sendo 10 (7,9%) antes e 19 (15,0%) após o tratamento com IVL. Ocorreu dilatação incompleta com balão de corte em 4 lesões, requerendo a terapia com IVL como estratégia de resgate. O número médio de cateteres de IVL utilizados por paciente foi de 1,18 ± 0,41. A mediana do número de pulsos de IVL aplicados por lesão foi de 80 (intervalo interquartil: 70–80), com 73,6% dos casos recebendo todos os 80 pulsos. Em quase metade dos casos, os balões de IVL atravessaram com sucesso as lesões calcificadas sem necessidade de pré-dilatação.

**Tabela 1:** – Características demográficas e clínicas basais (n = 104 pacientes)

Características	Todos os pacientes (n = 104)	Primeira coorte (n = 52)	Segunda coorte (n = 52)	Valor p
**Idade (anos)**	73,2 ± 10,2	72,6 ± 10,7	73,9 ± 9,77	0,5
**Sexo masculino**	79 (76)	39 (75)	40 (76,9)	0,8
**Diabetes mellitus**	46 (44,2)	20 (38,5)	26 (50)	0,2
**Hipertensão**	82 (78,8)	40 (76,9)	42 (80,8)	0,6
**Fibrilação atrial**	10 (9,6)	9 (17,3)	1 (1,9)	0,01
**Infarto do miocárdio prévio**	23 (22,1)	9 (17,3)	14 (26,9)	0,2
**CRM prévia**	14 (13,5)	6 (11,5)	8 (15,4)	0,5
**ICP prévia**	53 (51)	22 (42,3)	31 (59,6)	0,07
**Acidente vascular cerebral**	2 (1,9)	1 (1,9)	1 (1,9)	0,8
**Tabagismo (atual ou prévio)**	54 (51,9)	23 (44,2)	31 (59,6)	0,1
**Doença arterial coronariana multivascular**	81 (77,9)	38 (73,1)	43 (82,7)	0,3
**Doença renal crônica**				0,3
TFGe < 60 ml/min	35 (33,7)	21 (40,4)	14 (26,9)	0,1
TFGe < 30 ml/min	6 (5,8)	4 (7,7)	2 (3,8)	0,6
**Diálise**	4 (3,8)	2 (3,8)	2 (3,8)	1,00
**Fração de ejeção do ventrículo esquerdo (por ETT)**	61,0 ± 9,4	61,1 ± 8,92	61,0 ± 10,1	0,9
Fração de ejeção < 45%	7 (6,7)	4 (9,1)	3 (5,9)	0,7
**Apresentação clínica**				0,9
Síndrome coronariana crônica	39 (37,5)	20 (38,5)	19 (36,5)	1,0
SCA sem supradesnivelamento do segmento ST	59 (56,7)	29 (55,8)	30 (57,7)	1,0
SCA com supradesnivelamento do segmento ST	6 (5,8)	3 (5,8)	3 (5,8)	1,0

Os valores são apresentados como contagem (porcentagem) ou média (± desvio-padrão). CRM: cirurgia de revascularização do miocárdio; ICP: intervenção coronariana percutânea; SCA: síndrome coronariana aguda; TFGe: taxa de filtração glomerular estimada.

**Tabela 2:** – Características angiográficas basais e detalhes do procedimento (n = 104 pacientes; n = 127 lesões)

Características	Todas as lesões (n = 127 lesões)	Primeira coorte (n = 62 lesões)	Segunda coorte (n = 65 lesões)	Valor p
**Vaso-alvo da IVL**				0,1
Tronco da coronária esquerda, todos	14 (11,0)	8 (12,9)	6 (9,2)	0,7
Tronco da coronária esquerda não protegido	9 (7,1)	5 (8,1)	4 (6,2)	0,7
Artéria coronária descendente anterior esquerda	73 (57,5)	38 (61,3)	35 (53,8)	0,5
Artéria coronária circunflexa esquerda	21 (16,5)	5 (8,1)	16 (24,6)	0,02
Artéria coronária direita	18 (14,2)	10 (16,1)	8 (12,3)	0,7
Enxerto cirúrgico	1 (0,8)	1 (1,6)	0	0,9
**Lesões de bifurcação**	65 (51,1)	26 (41,9)	39 (60,0)	0,04
Lesões tipo B2 + C*	125 (98,4)	61 (98,4)	64 (98,5)	0,9
**Lesões com reestenose intra-stent**	15 (11,8)	8 (12,9)	7 (10,8)	0,9
**Calcificação grave na angiografia^**†**^**	93 (73,2)	49 (69,4)	50 (76,9)	0,3
**QCA**				
Diâmetro de referência do vaso, mm	3,13 ± 0,75	3,19 ± 0,81	3,09 ± 0,67	0,9
Diâmetro mínimo do lúmen, mm	1,22 ± 0,59	1,42 ± 0,68	1,29 ± 0,53	0,7
Extensão da lesão, mm	21,7 ± 11,7	20,5 ± 11,6	22,4 ± 11,3	0,3
Diâmetro da estenose, %	56,8 ± 17,0	54,9 ± 18,9	57,9 ± 15,7	0,5
Acesso vascular radial	61 (48)	33 (53,2)	28 (43,1)	0,2
**Calibre do introdutor**				0,6
6 French	101 (79,5)	50 (80,6)	51 (78,5)	0,9
7 French	25 (19,7)	12 (19,4)	13 (20,0)	1,0
8 French	1 (0,8)	0	1 (1,5)	1,0
**IVL inicial**	118 (92,9)	57 (91,9)	61 (93,8)	0,6
**IVL de resgate**	9 (7,1)	5 (8,1)	3 (6,2)	0,9
**Cateter de extensão de guia**	19 (15,0)	8 (12,9)	11 (16,9)	0,5
**Pré-dilatação antes da IVL**	65 (51,2)	32 (51,6)	33 (50,8)	0,9
**Pós-dilatação**	114 (89,8)	55 (91,7)	57 (87,7)	0,4
**Balões de corte**	29 (22,8)	14 (22,6)	15 (23,1)	1,0
Balões de corte antes da IVL	10 (7,9)	6 (9,7)	4 (6,2)	1,0
Balões de corte após a IVL	19 (15,0)	8 (12,9)	11 (16,9)	1,0
**Aterectomia rotacional**	8 (6,3)	5	3	0,4
**Pulsos de IVL aplicados**	73,3 ± 26,0	67,3 ± 19,6	81,5 ± 31,3	0,01
**Tamanho do cateter, n = 130**				0,02
2,5 mm	23 (17,7)	13 (20,0)	11 (15,4)	0,7
3,0 mm	49 (37,7)	18 (27,7)	31 (47,7)	0,02
3,5 mm	43 (33,1)	22 (33,8)	21 (32,3)	1,0
4,0 mm	15 (11,5)	12 (18,5)	3 (4,6)	0,02
**Número de stents**	1,8 ± 1,1	1,75 ± 0,97	1,88 ± 1,40	0,5
**ICP com DCB, todas as lesões**	12 (9,4)	3 (4,8)	9 (13,8)	0,1
**Imagem intravascular, por total de lesões**	99 (78,0)	53 (85,3)	46 (70,8)	0,07
IVUS	79 (62,2)	42 (67,7)	37 (56,9)	0,2
OCT	20 (15,7)	11 (11,7)	9 (13,8)	0,7
**Volume de contraste (ml)**	232 ± 97,8	216 ± 100	248 ± 93,2	0,08

Os valores são apresentados como contagem (porcentagem) ou média (± desvio-padrão). DCB: balão farmacológico; ICP: intervenção coronariana percutânea; IVL: litotripsia intravascular; IVUS: ultrassom intravascular; OCT: tomografia de coerência óptica; QCA: análise coronária quantitativa. * Classificação de Ellis.^18^ ^1^ Definida como densidades radiopacas observadas na ausência de movimento cardíaco, afetando tipicamente ambos os lados da parede arterial.

O desempenho procedimental e os desfechos intra-hospitalares estão resumidos nas Tabelas 3 e 4. O sucesso angiográfico foi alcançado em 96% dos casos. Complicações angiográficas graves relacionadas à IVL ocorreram em duas lesões (1,9%), incluindo um caso de perfuração coronária tipo 3 — associado à dilatação com balão não complacente após a aplicação dos pulsos de IVL — e um caso de fenômeno de fluxo lento. Todas as dissecções coronárias induzidas pela IVL (5 lesões) foram classificadas como grau inferior a D e resolvidas com sucesso mediante o implante de stent. A análise quantitativa coronária offline revelou um ganho luminal agudo médio de 1,55 ± 0,79 mm, com 96,8% das lesões apresentando estenose residual de diâmetro inferior a 30%. Não houve casos de reintervenção, infarto do miocárdio espontâneo ou oclusão aguda do vaso-alvo. No total, quatro pacientes sofreram infarto do miocárdio periprocedimento e um paciente faleceu devido a complicações decorrentes de perfuração coronária, resultando em uma incidência total de ECAM intra-hospitalares de 4,8%.

A primeira e a segunda coortes diferiram em algumas características angiográficas e procedimentais, conforme detalhado nas Tabelas 2 e 3. Na segunda coorte, a IVL foi utilizada com maior frequência em lesões de bifurcação e na artéria circunflexa, enquanto o uso de balões de IVL de maior calibre foi menos frequente. As complicações angiográficas diminuíram no período mais recente, embora a incidência de ECAM intra-hospitalares não tenha diferido entre as coortes temporais (Tabelas 3 e 4). A segunda metade do registro também demandou um período mais longo para incluir o mesmo número de pacientes, sugerindo um uso progressivamente mais seletivo da IVL ao longo do tempo (Figura 2). A Ilustração Central resume os principais achados do presente estudo.

**Tabela 3:** – Resultados angiográficos e do procedimento (n = 127 lesões)

	Todas as lesões (n=127 lesões)	Primeira coorte (n=62 lesões)	Segunda coorte (n=65 lesões)	Valor p
**Entrega bem-sucedida do dispositivo**				0,1
Stents, n = 115 lesões	115 (100)	59 (100)	56 (100)	
DCB, n = 12 lesões	12 (100)	3 (100)	9 (100)	
IVL	126 (99,2)	61 (98,4)	65 (100)	0,4
**QCA final intra-stent**				
Diâmetro mínimo do lúmen, mm	3,06 ±1,79	2,96 ± 0,59	2,86 ± 0,68	0,3
Ganho agudo, mm	1,55 ± 0,79	1,49 ± 0,90	1,62 ± 0,66	0,6
Estenose do diâmetro residual %	15,9 ± 9,12	16,23 ± 8,34	16,22 ± 9,21	0,9
< 50%	127 (100)	62 (100)	65 (100)	1,0
< 30%	123 (96,8)	61 (98,4)	62 (95,4)	1,0
Sucesso angiográfico*	122 (96,0)	60 (96,7)	62 (95,4)	1,0
**Complicações angiográficas**	10 (7,8)	9 (14,5)	1 (1,5)	0,01
Dissecções vasculares induzidas por IVL	5 (3,9)	5 (8,1)	0	0,02
Tipo B	3 (2,3)	3 (4,8)	0	0,1
Tipo C	2 (1,6)	2 (3,2)	0	0,2
Tipo ≥ D	0	0	0	-
Ruptura do balão de IVL	2 (1,6)	2 (3,2)	0	0,2
Perfuração	1 (0,8)	0	1 (1,5)	1,0
Fluxo lento	1 (0,8)	1 (1,6)	0	0,4
Oclusão do ramo lateral^†^	1 (0,8)	1 (1,6)	0	0,4
Oclusão do vaso principal	0	0	0	-
Aprisionamento do balão	0	0	0	-
**Fibrilação ventricular**	0	0	0	-

Os valores são apresentados como contagem (porcentagem) ou média (± desvio-padrão). DCB: balão farmacológico; IVL: litotripsia intravascular; QCA: análise coronária quantitativa. * Estenose residual < 30% após intervenção coronariana percutânea, pela QCA, em associação com fluxo Thrombolysis in Myocardial Infarction grau 3 e ausência de complicações angiográficas graves. ^†^ Após a aplicação dos pulsos de IVL e a subsequente insuflação de kissing balloon (KBI), foi restabelecido o fluxo Thrombolysis in Myocardial Infarction grau 3.

**Tabela 4:** – Desfechos clínicos intra-hospitalares (n = 104 pacientes; n = 127 lesões)

	Todos os pacientes (n=104)	Primeira coorte (n=52 pacientes) (n=62 lesões)	Segunda coorte (n=52 pacientes) (n=65 lesões)	Valor p
**Tempo mediano para alta hospitalar, dias**	2,0 (1,0–4,0)	2,0 (1,0–5,0)	2,0 (1,75–3,0)	0,9
**Morte cardíaca**	0,9 (1)	0	1.9 (1)	1,0
**Qualquer infarto do miocárdio**	4,8 (5)	5,8 (3)	3,8 (2)	1,0
Infarto do miocárdio com onda Q	0,9 (1)	0	1.9 (1)	1,0
**Revascularização do vaso-alvo**	0	0	0	1,0
**Qualquer trombose de stent**	0,9 (1)	0	1.9 (1)	1,0
**Qualquer ECAM**	4,8 (5)	5,8 (3)	3,8 (2)	1,0

Os valores são apresentados como porcentagem (contagem) ou mediana (intervalo interquartil). ECAM: eventos cardíacos adversos maiores; IVL: litotripsia intravascular.

**Figura 2:**
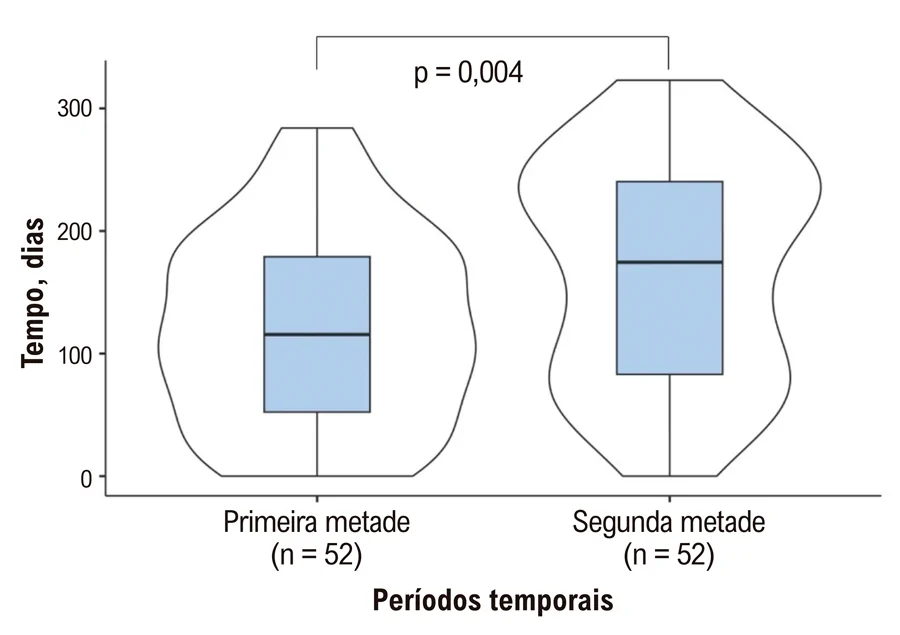
– Tempo médio de inclusão de pacientes para intervenção coronária percutânea assistida por litotripsia intravascular: 120 ± 72,8 dias na primeira metade e 168 ± 90,8 dias na segunda metade.

## Discussão

O presente registro prospectivo de centro único representa a experiência inicial de um centro cardiovascular de alto volume na incorporação da tecnologia de IVL para o tratamento de lesões coronárias calcificadas graves. O registro reflete resultados de vida real, abrangendo tanto síndromes coronárias crônicas quanto agudas. Os principais achados são: (1) A IVL demonstrou segurança e eficácia em uma população de alto risco, caracterizada por perfis clínicos e angiográficos complexos — incluindo lesões longas, bifurcações e tratamento do tronco da coronária esquerda não protegido — e por uma proporção significativa de pacientes admitidos com síndrome coronária aguda (incluindo infarto do miocárdio com supradesnivelamento do segmento ST), o que contrasta com estudos prospectivos anteriores.^13,14^ (2) Os operadores apresentaram uma curva de aprendizado curta com a tecnologia da IVL, evidenciada pela redução na incidência de complicações angiográficas no período mais recente, apenas alguns meses após a introdução da tecnologia. A segunda fase também abrangeu um período mais longo de inclusão de pacientes, refletindo um uso mais seletivo da IVL. (3) A estratégia preferencial foi o uso inicial da IVL, combinado com uma alta taxa de utilização de imagem intravascular para guiar e otimizar a ICP. (4) Dispositivos de modificação do cálcio — incluindo aterectomia rotacional, balões de corte e balões não complacentes — foram combinados com segurança à IVL para melhorar os resultados do procedimento.

Apesar de esta ter sido a primeira experiência de todos os operadores com a realização de ICP assistida por IVL, logo após a introdução da tecnologia no Brasil, alcançou-se uma taxa de sucesso angiográfico de 96%, acompanhada por baixas taxas de ECAM intra-hospitalares. Observou-se uma redução notável nas complicações angiográficas na segunda metade dos pacientes atendidos, o que provavelmente reflete a curva de aprendizado dos operadores com o dispositivo. Curiosamente, a segunda metade dos pacientes incluiu mais intervenções na artéria circunflexa, uma maior proporção de lesões em bifurcação, uso menos frequente de balões de IVL de 4,0 mm de diâmetro e menor incidência de dissecções coronárias antes do implante do stent. Ademais, esse grupo exigiu um período de inclusão mais prolongado. Esses achados sugerem que os operadores se tornaram mais confiantes e seletivos em relação à tecnologia à medida que sua experiência aumentava. É importante ressaltar que não foram observadas diferenças significativas nos desfechos clínicos entre a primeira e a segunda metade da coorte, reforçando o perfil de segurança da IVL ao longo de sua adoção. Embora todos os procedimentos tenham sido realizados por cardiologistas intervencionistas experientes, os resultados favoráveis observados também podem ser atribuídos à simplicidade e à facilidade de uso do sistema IVL. Ao utilizar o mesmo mecanismo de entrega via cateter que a angioplastia com balão convencional, a IVL provavelmente reduz a complexidade técnica e encurta a curva de aprendizado do operador.

O uso combinado da IVL com outros dispositivos de modificação do cálcio — como balões de corte, balões não complacentes e aterectomia rotacional — foi insuficientemente avaliado em estudos controlados e permanece pouco relatado em registros de vida real. É importante ressaltar que os mecanismos das técnicas de ateroablação diferem fundamentalmente dos da IVL, sendo necessárias investigações adicionais para determinar se essas abordagens proporcionam benefícios aditivos ou sinérgicos. Em nosso registro, o uso adjuvante da IVL com balões de corte/não complacentes e/ou aterectomia rotacional mostrou-se viável e seguro, podendo ter facilitado a transposição da lesão e a subsequente otimização do stent. No entanto, essas observações necessitam de validação em estudos clínicos controlados específicos. Em consonância com esse conceito, nosso grupo relatou recentemente que a ICP assistida por IVL associou-se a uma melhor expansão do stent e a uma maior área de secção transversal mínima final do stent em comparação com a ICP utilizando apenas aterectomia.^15^

A ICP complexa guiada por imagem intravascular é uma estratégia eficaz para otimizar o implante de stent e está associada a melhores desfechos clínicos.^16^ No presente registro, a imagem intravascular foi frequentemente utilizada na prática de rotina para auxiliar no planejamento e na otimização do procedimento durante a ICP complexa. Embora desfechos quantitativos derivados de imagem não tenham sido registrados sistematicamente como parte de um protocolo de imagem predefinido, essa prática pode ter facilitado uma avaliação mais precisa da lesão e um limiar mais baixo para a adoção de estratégias iniciais de modificação do cálcio, incluindo a IVL, além de ter contribuído para os desfechos procedimentais favoráveis observados. Esse padrão contrasta com vários registros nos quais a IVL foi empregada mais comumente como estratégia de resgate, frequentemente em resposta à expansão subótima do balão ou à subexpansão de stent.

O presente relato identificou apenas algumas dissecções angiográficas após a litotripsia realizada antes do implante de stent. Todas as dissecções observadas eram do tipo B ou C, com apenas um caso de fenômeno de fluxo lento. Vale ressaltar que a IVL foi realizada em conjunto com terapia com balão farmacológico (DCB) para tratar lesões *de novo* em vasos de pequeno calibre em 9 casos. Esses procedimentos resultaram em sucesso angiográfico, sem eventos clínicos adversos. Portanto, evitar o implante de stent no manejo de calcificação coronária grave em vasos de pequeno calibre pode representar uma indicação adicional para o tratamento com IVL. O ensaio prospectivo “IVL + DCB” (NCT 05625997) em andamento visa avaliar melhor essa estratégia.

A análise combinada em nível de paciente dos quatro estudos DISRUPT-CAD representa a maior série prospectiva de avaliação do tratamento de lesões coronárias com calcificação grave por meio de IVL, incluindo um total de 628 pacientes.^6^ Uma comparação numérica entre o nosso registro e as análises em nível de paciente dos estudos DISRUPT-CAD é apresentada na Tabela 5. No presente estudo, observamos desfechos clínicos e angiográficos intra-hospitalares semelhantes, com taxas de ECAM de 4,8%, em comparação com 6,5% no DISRUPT-CAD. É importante notar que os critérios para infarto do miocárdio periprocedimento diferiram entre os estudos, o que pode explicar as taxas mais baixas de infarto do miocárdio periprocedimento em nosso registro.^10,17^ Nossa coorte parece apresentar um perfil mais complexo em termos de características clínicas e procedimentais. Em suma, a série DISRUPT-CAD excluiu pacientes com síndromes coronárias agudas, lesões de tronco de coronária esquerda não protegido, doença renal grave e reestenose intra-stent. Por outro lado, nosso registro incluiu uma população de pacientes diversificada, sem critérios de exclusão específicos, relatando ICP assistida por IVL em pacientes em diálise, casos de reestenose intra-stent, uso de DCB para lesões *de novo* e utilização de métodos combinados para modificar lesões calcificadas. Os desfechos angiográficos e clínicos favoráveis e comparáveis entre os dois estudos sugerem o potencial de expansão das indicações *on-label* atuais para a IVL coronária.

**Tabela 5:** – Comparação das análises combinadas em nível de paciente dos estudos DISRUPT CAD I-IV com o presente estudo

Características	Presente estudo (n = 104)	DISRUPT CAD I-IV (n = 628)
**Idade (anos)**	73,2 ±10,2	71,8 ± 8,9
**Sexo masculino**	79 (76)	484 (77,1)
**Diabetes mellitus**	46 (44,2)	241 (38,4)
**Hipertensão**	82 (78,8)	539 (85,8)
**Infarto do miocárdio prévio**	23 (22,1)	137 (21,8)
**Cirurgia de revascularização do miocárdio prévia**	14 (13,5)	60 (9,6)
**Insuficiência renal grave (TFGe <30 ml/min/1,73 m^**2**^)**	6 (5,8)	Excluído
**Síndromes coronarianas agudas**	65 (62,5)	Excluído
**Imagem intravascular, %**	78	54
**Extensão da lesão (mm)**	21,7 ± 11,7	24,4 ± 11,5
**Envolvimento de ramo lateral**	65 (51,1)	190 (30,3)
**Calcificação coronariana grave na angiografia**	93 (73,2)	609 (97,0)
**ICP assistida por IVL em tronco da coronária esquerda não protegido**	9 (7,1)	Excluído
**Desfechos angiográficos finais intra-stent**		
Ganho agudo, mm	1,55 ± 0,79	1,68 ± 0,47
Diâmetro mínimo do lúmen, mm	3,06 ± 1,79	2,75 ± 0,44
Estenose residual < 30%	96,8	95,7 (97,7–99,6)
**Eventos cardíacos adversos maiores, %**	4,8	6,5 (4,7–8,8)
Complicações angiográficas graves,* %	1,9	2,1

Os valores são apresentados como contagem (porcentagem) ou média (± desvio-padrão). ICP: intervenção coronariana percutânea; IVL: litotripsia intravascular; TFGe: taxa de filtração glomerular estimada. * Dissecções ≥ grau D, perfurações, fechamento abrupto e fluxo lento/no-reflow.

O presente estudo possui algumas limitações que devem ser destacadas. Trata-se de um estudo prospectivo, unicêntrico e não randomizado; portanto, não podemos descartar a possibilidade de viés de seleção, e os desfechos favoráveis aqui observados devem ser interpretados com cautela. No entanto, os resultados somam-se ao crescente corpo de dados globais sobre o uso da litotripsia coronária em diferentes populações, o que é fundamental para qualquer tecnologia recente. Métodos combinados com a IVL, como o uso de outros dispositivos de ablação, demonstraram segurança nesta pequena série de casos; contudo, essa abordagem requer confirmação de sua eficácia em ensaios clínicos controlados de maior porte. Todos os procedimentos foram realizados por operadores experientes em ICP complexa, com ampla utilização de imagem intravascular, fator que pode ter contribuído para os desfechos favoráveis, independentemente da terapia com IVL. Entretanto, dada a ausência de desfechos quantitativos derivados de imagens intravasculares coletados de forma sistemática e a falta de um grupo controle para comparação, qualquer associação com os desfechos favoráveis observados permanece apenas como uma hipótese a ser investigada. Vale ressaltar que esta foi a primeira vez que todos os operadores utilizaram a litotripsia coronária, a qual se mostrou uma ferramenta prática, embora o acompanhamento a longo prazo seja essencial para avaliar a persistência da eficácia intra-hospitalar observada. Por fim, a comparação entre a nossa população e outros registros é necessariamente especulativa.

## Conclusões

O presente estudo apresenta a experiência inicial com ICP assistida por IVL para o tratamento de lesões coronárias gravemente calcificadas em um centro cardiovascular de alto volume. Ao longo do tempo, observou-se uma redução nas complicações angiográficas — particularmente dissecções — entre a primeira e a segunda coorte, o que sugere uma curva de aprendizado e adaptação curta e uma crescente familiaridade do operador com a técnica. A segunda coorte também exigiu um período de inclusão mais longo para atingir um tamanho amostral semelhante, o que pode refletir uma utilização progressivamente mais seletiva da IVL à medida que a experiência institucional amadureceu. É importante ressaltar que os eventos clínicos adversos intra-hospitalares foram pouco frequentes e não diferiram entre as coortes temporais. Em conjunto, nossos achados corroboram a segurança e a eficácia da ICP assistida por IVL na prática de rotina durante a fase inicial de adoção, ao mesmo tempo em que destacam a relevância da experiência do operador e da seleção de casos.
