# Supplementary material for: Incorporação da Litotripsia Intracoronária na Prática Clínica: Uma Análise Comparativa Temporal de Segurança, Eficácia e Curva de Aprendizado
Source: Arq Bras Cardiol. 2026 Sep 8;123(8):e20250783. [Article in Portuguese] doi: 10.36660/abc.20250783 (PMC13671136; doi:10.36660/abc.20250783)
Supplement: Translation

# Translation: Incorporating Intracoronary Lithotripsy into Daily Practice: A Temporal Comparative Analysis of Safety, Efficacy, and Learning Curve

**DOI:** 10.36660/abc.20250783i

**Authors:** Guy F. A. Prado, Stefano Garzon, José Mariani, Willterson Bandeira, Felipe Mateus Teixeira Bezerra, Breno Oliveira Almeida, Gustavo Neves de Araujo, Ana Claudia Conrado, João Silveira, Emanuele Barbato, Flavio Ribichini, Pedro Alves Lemos

**Affiliations:** 1 Brasil Hospital Israelita Albert Einstein, São Paulo, SP – Brasil; 2 Itália University of Rome La Sapienza - Department of Clinical and Molecular Medicine, Rome – Itália; 3 Itália Universita degli Studi di Verona Scuola di Medicina e Chirurgia – Cardiology, Verona – Itália; 4 Brasil Unimed Grande Florianópolis, Florianópolis, SC – Brasil; 5 Portugal Centro Hospitalar Universitario do Porto EPE, Porto – Portugal

**Keywords:** Lithotripsy, Learning Curve, Interventional Ultrasonography, Percutaneous Coronary Intervention

## Abstract

**Background:**

Intravascular lithotripsy (IVL) has emerged as a groundbreaking technology for treating calcified coronary lesions.

**Objective:**

To assess whether case selection and interventional performance with IVL have evolved over time after its incorporation into daily practice.

**Methods:**

We prospectively included 104 consecutive patients with 127 calcified coronary lesions treated with IVL. The cohort was divided into two temporal groups (52 patients each) based on the order of patient inclusion. Clinical data and procedural performance were compared between the groups. All statistical tests were two-sided, with a significance level of 0.05.

**Results:**

The mean age was 73.2 ± 10.2 years, and 62.5% of patients presented with acute coronary syndromes. Severe calcification and type B2/C lesions were identified in 73.2% and 98.4%, respectively. Upfront IVL was performed in 92.9% of lesions, intravascular imaging was used in 78%, rotational atherectomy in 6.3%, and angiographic success was achieved in 96.0%. Compared with the first cohort, the latter cohort had more bifurcation lesions (60.0% vs. 41.9%; p = 0.04), more interventions in the circumflex artery (24.6% vs. 8.1%; p = 0.02), and a lower use of 4.0-mm diameter balloons (4.6% vs. 18.5%; p = 0.02). The latter cohort spanned a longer inclusion period (168 ± 90.8 vs. 120 ± 72.8 days; p = 0.004) and had fewer angiographic complications (1.5% vs. 14.5%; p = 0.01), whereas in-hospital major adverse cardiac events did not differ (3.8% vs. 5.8%; p = 1.0).

**Conclusion:**

IVL is an effective modality for coronary calcium modification. Temporal changes in case selection and interventional performance may reflect a learning-adaptation curve.

## Introduction

The prevalence of calcified coronary lesions in patients undergoing percutaneous coronary intervention (PCI) is progressively increasing due to advanced age and the presence of multiple comorbidities.^1^ Severe coronary calcification poses a significant challenge to PCI, as it restricts the navigation and delivery of intracoronary devices, prolongs procedure time, hinders optimal stent expansion, and increases the risk of vessel dissection or rupture. Additionally, manipulating coronary stents through calcified segments may damage the polymeric coating and the metallic platform, as well as the delivery system. Altogether, the presence of severe calcification leads to an increased risk not only of acute complications but also of worse long-term clinical outcomes.^2^

Various techniques and devices have been developed to facilitate PCI of calcified lesions, including non-compliant and scoring balloons, rotational and orbital atherectomy, and excimer laser therapy. The selection of the optimal therapeutic strategy is usually guided by a structured, consensus-informed workflow for the contemporary management of calcified coronary lesions.^3^ However, several limitations still exist, making this specific lesion subset a most challenging one, often requiring a high level of operator expertise.

Intravascular lithotripsy (IVL) was developed as a dedicated tool for treating calcified vessels. It incorporates the same physical properties as extracorporeal lithotripsy for nephrolithiasis, using electrical energy to generate sonic waves that selectively fracture both superficial and deep calcium in the vessel wall through a semi-compliant balloon at low pressure (4 to 6 atmospheres).^4^ The technology has been assessed in multiple studies and has progressively entered clinical practice following regulatory clearance in different jurisdictions, supported by prospective trials and real-world registries.^5, 6^

The performance of interventional technologies may diverge from that observed in clinical trials when translated to routine practice, particularly in high-complexity settings such as severely calcified coronary lesions. Furthermore, every new technology in the medical field may entail a learning curve. This context motivated the present study, which aimed to evaluate the early real-world experience with IVL-assisted PCI after the incorporation of this technology into daily practice at a high-volume cardiovascular center. Specifically, we sought to assess the safety and efficacy of IVL in consecutive patients with calcified coronary lesions and to explore whether case selection and procedural performance changed over time, as a marker of a potential learning-adaptation curve.

## Methods

### Patients and study design

Upon its approval in Brazil in June 2022, IVL was incorporated into our center’s routine interventional armamentarium. The present study includes all consecutive patients treated with the new technology at the institution, from the very initial case, which was the first case performed in Brazil (Figure 1), until January 2024. All patients admitted with either chronic or acute coronary syndrome who received IVL treatment for severely calcified coronary lesions were included in the study as part of a prospective single-center registry. Additionally, to assess the operators’ proficiency, the cohort was divided into two groups of similar size based on the temporal order of patient inclusion (first and second halves). The groups were compared with respect to clinical and procedural characteristics and the occurrence of adverse outcomes. The decision to perform PCI was left to the discretion of the operators, clinical cardiologists, or institutional heart team, who adhered to current international guidelines for appropriate revascularization. The indication for IVL, whether as an upfront or bailout strategy, for example, after failure of high-pressure non-compliant balloon dilatation, as well as its combination with other calcium-modifying devices, such as rotational atherectomy or cutting balloons, generally followed contemporary consensus-informed recommendations for the management of severe calcified coronary lesions.^3^ However, given the pragmatic design of this prospective registry and the absence of a dedicated study protocol, the final decision to use IVL was left to operator discretion, reflecting daily practice in a high-volume catheterization laboratory.

**Figure 1:** – The first patient treated in Brazil on June 13, 2022, with PCI assisted by intravascular lithotripsy for a successful bailout procedure. The upper panel in (A) shows IVUS imaging of an arc of calcium in the LAD while the lower panel in (A) depicts angiography of a critical calcified lesion in the LAD. In (B), the upper panel shows IVUS imaging illustrating the unchanged calcium after non-compliant balloon dilatation at high pressure in the LAD, while the lower panel presents angiography of an incomplete non-compliant balloon dilatation at high pressure at the same lesion. In (C), the upper panel shows narrow white marks indicating the cracking of the calcium identified by IVUS in the LAD, while the lower panel features angiography of the lithotripsy balloon (3.5 × 12mm) releasing 80 (8 × 10) pulses of acoustic waves at the same lesion. Finally, in (D), the upper panel reveals the final result after the implantation of two drug-eluting stents assessed by IVUS, while the lower panel shows the final angiography result. The LAD MLA before and after the procedure was 2.36 mm2 and 11.32 mm2, respectively. IVUS: intravascular ultrasound; LAD: left anterior descending artery, MLA: minimum lumen area.

The study adhered to the principles of the Declaration of Helsinki and received approval from the institutional review board and ethics committee of Albert Einstein Israelite Hospital. All patients were approached for informed consent, either in person or by email; for those who did not respond, the local ethics committee waived the requirement for informed consent regarding the collection, analysis, and publication of this non-interventional study analysis.

### Coronary intravascular lithotripsy system

The coronary IVL (Shockwave Medical, Santa Clara, CA, USA) catheter is a rapid exchange catheter that contains two lithotripsy emitters integrated into a semi-compliant coronary balloon (available in 2.5- to 4.0-mm diameters and 12 mm in length). During the procedure, the IVL balloon filled with diluted contrast is inflated at the target segment (4 atmospheres) followed by the local application of sonic pressure waves by emitters, which create a circumferential field effect aiming to selectively fracture calcium and alter vessel compliance. The generator is preprogrammed to deliver bursts of 10 pulses (1 pulse/s) for a maximum of 80 pulses per catheter.^7^ For this initial series of patients, the new IVL catheter C2 plus comprising 120 pulses was unavailable.

### Percutaneous coronary intervention

All procedures were performed exclusively by five operators experienced in complex PCI, and patients were given at least 100 mg of acetylsalicylic acid before the procedure. For those who were not under dual antiplatelet therapy, a loading dose, preferably of prasugrel or ticagrelor, was given after the PCI or, alternatively, clopidogrel. Patients were advised to continue dual-antiplatelet therapy for a minimum of 6 months for stable coronary disease and 1 year for acute coronary syndromes in accordance with the current guidelines.^8^ Given the complex nature of these procedures, the use of intravascular ultrasound (IVUS) or optical coherence tomography (OCT) to guide PCI was strongly encouraged; nevertheless, imaging utilization was determined by operator discretion.

### Endpoints and definitions

To assess clinical and procedural performance, patients were monitored for the primary endpoint of in-hospital major adverse cardiac events (MACE), defined as the composite of cardiac death, myocardial infarction, and target vessel revascularization. The study also collected data on angiographic or device-related complications, including vessel perforation, acute vessel closure, acute side-branch closure, no-reflow, slow flow, IVL balloon entrapment, IVL balloon rupture, coronary dissections, and ventricular fibrillation. Severe coronary complications were defined as (i) type D, E, or F dissections; (ii) vessel closure; (iii) any coronary perforation; and (iv) slow flow/no-reflow. Angiographic success was defined as final residual stenosis after PCI < 30% by quantitative coronary analysis (QCA), in combination with Thrombolysis in Myocardial Infarction (TIMI) grade 3 flow and absence of serious angiographic complications, adapted from previous consensus definitions.^9^

Myocardial infarction was defined according to the Fourth Universal Definition of Myocardial Infarction.^10^ Stent thrombosis was defined in agreement with the Academic Research Consortium guidelines.^11^ The American College of Cardiology/American Heart Association (ACC/AHA) lesion morphology classification was adopted.^12^

Severe angiographic calcification was defined as radio-opacities observed without cardiac motion, typically affecting both sides of the arterial lumen. Severe calcification assessed by IVUS or OCT was defined as calcification exceeding 270° or 180° of the vessel contours, respectively, with extensions of at least 5 mm or calcium thickness visualized by OCT greater than 500 μm.

### Statistical analysis

Categorical variables are presented as counts (percentages). Continuous variables are presented as mean ± standard deviation when normally distributed and as median (interquartile range) otherwise. Normality was assessed using the Shapiro–Wilk test and visual inspection of histograms and Q–Q plots. Between-group comparisons for categorical variables were performed using the chi-square test or Fisher’s exact test, as appropriate. Continuous variables were compared between independent groups using the unpaired Student’s t-test for approximately normally distributed data and the Mann–Whitney U test for non-normally distributed data. As outcomes were limited to events occurring during the index hospitalization, no time-to-event analyses (Kaplan–Meier) were performed; event rates were compared between temporal cohorts using Fisher’s exact test. Sample size calculations were not performed because the study was derived from a single-center registry. All analyses were conducted using Jamovi^®^ (version 2.5.7; The Jamovi Project, Sydney, Australia). All hypothesis tests were two-sided, with a significance level of 0.05.

## Results

Overall, 104 patients with 127 lesions were included. The mean age was 73.2 ± 10.2 years, 76% were male, 62.5% had acute coronary syndrome, and 44.2% had diabetes (Table 1). Multivessel coronary artery disease was observed in 77.9% of patients, whereas type B2/C lesions and severe fluoroscopic calcification were identified in 73.2% and 98.4% of cases, respectively (Table 2). The mean lesion length was 21.7 ± 11.7 mm, with bifurcation involvement in 51.1% and unprotected left main disease in 7.1% of lesions (Table 2). IVL was used as the initial treatment strategy in 92.9% of lesions, with the remainder treated as a bailout after incomplete balloon pre-dilatation. Intravascular imaging was utilized in 78.0% of cases. Rotational atherectomy was performed in 8 (6.3%) lesions before IVL therapy as an adjunctive treatment to facilitate IVL balloon crossing. Cutting balloons were used in 29 lesions (22.8%), with 10 (7.9%) used before and 19 (15.0%) after IVL treatment. Notably, incomplete cutting balloon dilatation occurred in 4 lesions, requiring IVL therapy as a bailout strategy. The average number of IVL catheters used per patient was 1.18 ± 0.41. The median number of IVL pulses delivered per lesion was 80 (interquartile range: 70–80), with 73.6% of cases receiving all 80 pulses. In nearly half of the cases, IVL balloons successfully crossed the calcified lesions without requiring pre-dilation.

**Table 1:** –Baseline and clinical demographics (n = 104 patients)

Characteristics	All patients (n = 104)	Initial cohort (n = 52)	Latter cohort (n = 52)	p-value
**Age (years)**	73.2 ± 10.2	72.6 ± 10.7	73.9 ± 9.77	0.5
**Male**	79 (76)	39 (75)	40 (76.9)	0.8
**Diabetes mellitus**	46 (44.2)	20 (38.5)	26 (50)	0.2
**Hypertension**	82 (78.8)	40 (76.9)	42 (80.8)	0.6
**Atrial fibrillation**	10 (9.6)	9 (17.3)	1 (1.9)	0.01
**Prior myocardial infarction**	23 (22.1)	9 (17.3)	14 (26.9)	0.2
**Prior CABG**	14 (13.5)	6 (11.5)	8 (15.4)	0.5
**Prior PCI**	53 (51)	22 (42.3)	31 (59.6)	0.07
**Stroke**	2 (1.9)	1 (1.9)	1 (1.9)	0.8
**Smoker (current or former)**	54 (51.9)	23 (44.2)	31 (59.6)	0.1
**Multivessel coronary artery disease**	81 (77.9)	38 (73.1)	43 (82.7)	0.3
**Chronic kidney disease**				0.3
eGFR < 60 ml/min	35 (33.7)	21 (40.4)	14 (26.9)	0.1
eGFR < 30 ml/min	6 (5.8)	4 (7.7)	2 (3.8)	0.6
**Dialysis**	4 (3.8)	2 (3.8)	2 (3.8)	1.00
**Left ventricular ejection fraction (by TTE)**	61.0 ± 9.4	61.1 ± 8.92	61.0 ± 10.1	0.9
Ejection fraction < 45%	7 (6.7)	4 (9.1)	3 (5.9)	0.7
**Clinical presentation**				0.9
Chronic coronary syndrome	39 (37.5)	20 (38.5)	19 (36.5)	1.0
NSTEACS	59 (56.7)	29 (55.8)	30 (57.7)	1.0
STEACS	6 (5.8)	3 (5.8)	3 (5.8)	1.0

Numbers are counts (percentages) or means (± standard deviations). CABG: coronary artery bypass graft; eGFR: estimated glomerular filtration rate; NSTEACS: non-ST-elevation acute coronary syndrome; STEACS: ST-elevation acute coronary syndrome; PCI: percutaneous coronary intervention; TTE: transthoracic echocardiography.

**Table 2:** – Baseline angiographic characteristics and procedural details (n = 104 patients; n = 127 lesions)

Characteristics	All lesions (n = 127 lesions)	Initial cohort (n = 62 lesions)	Latter cohort (n = 65 lesions)	p-value
**IVL target vessel**				0.1
Left main, all	14 (11.0)	8 (12.9)	6 (9.2)	0.7
Left main, unprotected	9 (7.1)	5 (8.1)	4 (6.2)	0.7
Left anterior descending coronary artery	73 (57.5)	38 (61.3)	35 (53.8)	0.5
Left circumflex coronary artery	21 (16.5)	5 (8.1)	16 (24.6)	0.02
Right coronary artery	18 (14.2)	10 (16.1)	8 (12.3)	0.7
Surgical graft	1 (0.8)	1 (1.6)	0	0.9
**Bifurcation lesions**	65 (51.1)	26 (41.9)	39 (60.0)	0.04
Lesion type B2 + C*	125 (98.4)	61 (98.4)	64 (98.5)	0.9
**ISR lesion**	15 (11.8)	8 (12.9)	7 (10.8)	0.9
**Angiographic severe calcification^**†**^**	93 (73.2)	49 (69.4)	50 (76.9)	0.3
**Quantitative coronary analysis**				
Reference vessel diameter, mm	3.13 ± 0.75	3.19 ± 0.81	3.09 ± 0.67	0.9
Minimum lumen diameter, mm	1.22 ± 0.59	1.42 ± 0.68	1.29 ± 0.53	0.7
Lesion length, mm	21.7 ± 11.7	20.5 ± 11.6	22.4 ± 11.3	0.3
Diameter of stenosis, %	56.8 ± 17.0	54.9 ± 18.9	57.9 ± 15.7	0.5
Radial vascular access	61 (48)	33 (53.2)	28 (43.1)	0.2
**Sheath size**				0.6
6 French	101 (79.5)	50 (80.6)	51 (78.5)	0.9
7 French	25 (19.7)	12 (19.4)	13 (20.0)	1.0
8 French	1 (0.8)	0	1 (1.5)	1.0
**Upfront IVL**	118 (92.9)	57 (91.9)	61 (93.8)	0.6
**Bailout IVL**	9 (7.1)	5 (8.1)	3 (6.2)	0.9
**Guide extension catheter**	19 (15.0)	8 (12.9)	11 (16.9)	0.5
**Pre-dilation prior IVL**	65 (51.2)	32 (51.6)	33 (50.8)	0.9
**Post-dilation**	114 (89.8)	55 (91.7)	57 (87.7)	0.4
**Cutting balloons**	29 (22.8)	14 (22.6)	15 (23.1)	1.0
Cutting balloons before IVL	10 (7.9)	6 (9.7)	4 (6.2)	1.0
Cutting balloons after IVL	19 (15.0)	8 (12.9)	11 (16.9)	1.0
**Rotational atherectomy**	8 (6.3)	5	3	0.4
**IVL pulses released**	73.3 ± 26.0	67.3 ± 19.6	81.5 ± 31.3	0.01
**Catheter size, n = 130**				0.02
2.5 mm	23 (17.7)	13 (20.0)	11 (15.4)	0.7
3.0 mm	49 (37.7)	18 (27.7)	31 (47.7)	0.02
3.5 mm	43 (33.1)	22 (33.8)	21 (32.3)	1.0
4.0 mm	15 (11.5)	12 (18.5)	3 (4.6)	0.02
**Number of stents**	1.8 ± 1.1	1.75 ± 0.97	1.88 ± 1.40	0.5
**DCB-based PCI, all lesions**	12 (9.4)	3 (4.8)	9 (13.8)	0.1
**Intravascular imaging, per total lesions**	99 (78.0)	53 (85.3)	46 (70.8)	0.07
IVUS	79 (62.2)	42 (67.7)	37 (56.9)	0.2
OCT	20 (15.7)	11 (11.7)	9 (13.8)	0.7
**Contrast volume (ml)**	232 ± 97.8	216 ± 100	248 ± 93.2	0.08

Numbers are counts (percentages) and means (± standard deviations). DCB: drug-coated balloon; ISR: in-stent restenosis; IVL: intravascular lithotripsy; IVUS: intravascular ultrasound; OCT: optical coherence tomography; PCI: percutaneous coronary intervention. * Ellis classification.^1^ ^8^^†^ Defined as radiopaque densities observed without cardiac motion, typically affecting both sides of the arterial wall.

Procedure performance and in-hospital outcomes are summarized in Tables 3 and 4. Angiographic success was achieved in 96% of cases. Severe IVL-related angiographic complications occurred in two lesions (1.9%), including one case of type 3 coronary perforation, which was associated with non-compliant balloon dilatation following IVL pulse delivery, and one case of slow-flow phenomenon. All IVL-induced coronary dissections (5 lesions) were classified below grade D and were successfully resolved with stenting. Offline quantitative coronary analysis revealed a mean acute luminal gain of 1.55 ± 0.79 mm, with 96.8% of lesions demonstrating residual diameter stenosis of less than 30%. There were no cases of repeated intervention, spontaneous myocardial infarction, or acute target vessel closure. In total, four patients had a peri-procedural myocardial infarction, and one patient died due to complications after coronary perforation, yielding a total incidence of in-hospital MACE of 4.8%.

**Table 3:** – Procedural and angiographic outcomes (n = 127 lesions))

	All lesions (n=127 lesions)	Initial cohort (n=62 lesions)	Latter cohort (n=65 lesions)	p-value
**Successful device delivery**				0.1
Stents, n = 115 lesions	115 (100)	59 (100)	56 (100)	
DCB, n = 12 lesions	12 (100)	3 (100)	9 (100)	
IVL	126 (99.2)	61 (98.4)	65 (100)	0.4
**Final in-stent angiographic QCA**				
Minimum lumen diameter, mm	3.06 ±1.79	2.96 ± 0.59	2.86 ± 0.68	0.3
Acute gain, mm	1.55 ± 0.79	1.49 ± 0.90	1.62 ± 0.66	0.6
Residual diameter stenosis %	15.9 ± 9.12	16.23 ± 8.34	16.22 ± 9.21	0.9
< 50%	127 (100)	62 (100)	65 (100)	1.0
< 30%	123 (96.8)	61 (98.4)	62 (95.4)	1.0
Angiographic success*	122 (96.0)	60 (96.7)	62 (95.4)	1.0
**Angiographic complications**	10 (7.8)	9 (14.5)	1 (1.5)	0.01
IVL-induced vessel dissections	5 (3.9)	5 (8.1)	0	0.02
Type B	3 (2.3)	3 (4.8)	0	0.1
Type C	2 (1.6)	2 (3.2)	0	0.2
Type ≥ D	0	0	0	-
IVL balloon rupture	2 (1.6)	2 (3.2)	0	0.2
Perforation	1 (0.8)	0	1 (1.5)	1.0
Slow flow	1 (0.8)	1 (1.6)	0	0.4
Lateral branch closure ^†^	1 (0.8)	1 (1.6)	0	0.4
Main vessel closure	0	0	0	-
Balloon entrapment	0	0	0	-
**Ventricular fibrillation**	0	0	0	-

Numbers are counts (percentages) and means (± standard deviations). DCB: drug-coated balloon; IVL: intravascular lithotripsy; QCA: quantitative coronary analysis. * Residual stenosis after percutaneous coronary intervention of less than 30% by quantitative coronary analysis in combination with Thrombolysis in Myocardial Infarction flow 3 without serious angiographic complications. ^†^ After IVL pulse delivery and subsequent kissing-balloon inflation (KBI), Thrombolysis in Myocardial Infarction grade 3 flow was restored.

**Table 4:** – In-hospital clinical outcomes (n = 104 patients; n = 127 lesions)

	All patients (n=104)	Initial cohort (n=52 patients) (n=62 lesions)	Latter cohort (n=52 patients) (n=65 lesions)	p-value
**Median time to hospital discharge, days**	2.0 (1.0–4.0)	2.0 (1.0–5.0)	2.0 (1.75–3.0)	0.9
**Cardiac death**	0.9 (1)	0	1.9 (1)	1.0
**Any myocardial infarction**	4.8 (5)	5.8 (3)	3.8 (2)	1.0
Q-wave myocardial infarction	0.9 (1)	0	1.9 (1)	1.0
**Target vessel revascularization**	0	0	0	1.0
**Stent thrombosis, any**	0.9 (1)	0	1 (1.9)	1.0
**Any MACE**	4.8 (5)	5.8 (3)	3.8 (2)	1.0

Numbers are percentages (counts) or medians (interquartile range). IVL: intravascular lithotripsy; MACE: major adverse cardiac events.

The initial and latter cohorts differed in selected angiographic and procedural characteristics, as detailed in Tables 2 and 3. In the latter cohort, IVL was more frequently used in bifurcation lesions and in the circumflex artery, whereas the use of larger IVL balloons was less frequent. Angiographic complications decreased in the latter period, although in-hospital MACE did not differ between temporal cohorts (Tables 3 and 4). The latter half of the registry also required a longer period to enroll the same number of patients, suggesting progressively more selective IVL use over time (Figure 2). The Central Illustration summarizes the main findings of the study.

**Figure 2:** – Mean patient enrolment time for percutaneous coronary intervention assisted by intravascular lithotripsy: 120 ± 72.8 days in the first half and 168 ± 90.8 days in the second half.

## Discussion

This single-center prospective registry represents the early experience at a high-volume cardiovascular center incorporating IVL technology for treating severe calcified coronary lesions. The registry reflects real-world outcomes including both chronic and acute coronary syndromes. The key findings are as follows: (1) IVL demonstrated safety and efficacy in a high-risk population characterized by complex clinical and angiographic profiles, including long lesions, bifurcations, and unprotected left main treatments, and a substantial proportion of patients presenting with acute coronary syndromes on admission, including STEMI, which contrasts with previous prospective studies.^13,14^ (2) Operators appeared to experience a short learning curve with IVL technology, as evidenced by a reduced incidence of angiographic complications during the latter period, only a few months after the introduction of the technology. The latter phase also covered a longer patient inclusion time, reflecting more selective IVL use. (3) Upfront IVL, combined with high utilization of intravascular imaging to guide and optimize PCI, was the preferred strategy. (4) Calcium modification devices, including rotational atherectomy, cutting balloons, and non-compliant balloons, were safely combined with IVL to enhance procedural outcomes.

Despite this being the first experience of all operators performing PCI with IVL assistance, starting the usage just after the introduction of the technology in Brazil, an angiographic success rate of 96% was achieved, accompanied by low rates of in-hospital MACE. A notable reduction in angiographic complications was observed in the second half of the patients treated, likely reflecting the operator learning curve with the device. Interestingly, the second half of patients included more interventions in the circumflex artery, a higher proportion of bifurcation lesions, less frequent use of 4.0-mm diameter IVL balloons, and a lower incidence of coronary dissections prior to stent implantation. Additionally, this group required a more extended enrolment period. These findings suggest that operators became more confident and selective with the technology as their experience increased. Importantly, no significant differences in clinical outcomes were observed between the first and second halves of the cohort, reinforcing the safety profile of IVL throughout its adoption. Although experienced interventional cardiologists conducted all procedures, the favorable outcomes observed may also be attributed to the simplicity and user-friendliness of the IVL system. By using the same catheter delivery mechanism as traditional balloon angioplasty, IVL likely decreases technical complexity and shortens the operator learning curve.

The combined use of IVL with other calcium-modifying devices, such as cutting balloons, non-compliant balloons, and rotational atherectomy, has been insufficiently examined in controlled trials and remains sparsely reported in real-world registries. Importantly, the mechanisms of atheroablative techniques differ fundamentally from IVL, and whether these approaches provide additive or synergistic benefits warrants further investigation. In our registry, the adjunctive use of IVL with cutting/non-compliant balloons and/or rotational atherectomy appeared feasible and safe and may have facilitated lesion crossing and subsequent stent optimization. Nevertheless, these observations require validation in dedicated controlled clinical studies. In support of this concept, our group recently reported that IVL-assisted PCI was associated with improved stent expansion and larger final minimal stent cross-sectional area compared with PCI using atherectomy alone.^15^

Intravascular imaging-guided complex PCI is an effective strategy for optimizing stent implantation and is associated with improved clinical outcomes.^16^ In the present registry, intravascular imaging was frequently used in routine practice to support procedural planning and optimization during complex PCI. Although imaging-derived quantitative endpoints were not systematically captured as part of a prespecified imaging protocol, this practice may have facilitated more accurate lesion assessment, a lower threshold for adopting upfront calcium-modification strategies, including IVL, and may have contributed to the favorable procedural outcomes observed. This pattern contrasts with several registries in which IVL is more commonly employed as a bailout strategy, often in response to suboptimal balloon expansion or stent underexpansion.

This report found only a few angiographic dissections after lithotripsy prior to stent implantation. All dissections observed were type B or C, with only one case of slow-flow phenomenon. Notably, IVL was performed in conjunction with drug-coated balloon (DCB) therapy to treat de novo lesions in small vessels in nine cases. These procedures resulted in angiographic success with no adverse clinical events. Therefore, avoiding stent implantation to manage severe coronary calcification in small vessels may represent an additional indication for IVL treatment. The ongoing prospective trial “IVL + DCB” (NCT 05625997) aims to evaluate this strategy further.

The patient-level pooled analysis of the four DISRUPT-CAD studies represents the most extensive prospective series evaluating the treatment of severe coronary calcified lesions with IVL, enrolling a total of 628 patients.^6^ A numerical comparison between our registry and the patient-level analyses from the DISRUPT-CAD studies is presented in Table 5. In the current study, we observed similar in-hospital clinical and angiographic outcomes, with rates of MACE of 4.8% compared to 6.5% in DISRUPT-CAD. It is important to note that the criteria for periprocedural myocardial infarction differed between the studies, which may explain the lower rates of periprocedural myocardial infarction in our registry.^10,17^ Our cohort appears to exhibit a more complex profile in terms of clinical and procedural characteristics. In summary, the DISRUPT-CAD series excluded patients with acute coronary syndromes, unprotected left main lesions, severe kidney disease, and in-stent restenosis. Conversely, our registry included a diverse patient population without specific exclusion criteria, reporting PCI assisted by IVL in dialysis patients, cases of in-stent restenosis, DCB for de novo lesions, and the use of combined methods to modify calcified lesions. The favorable and comparable angiographic and clinical outcomes between the two studies suggest the potential for expanding the current on-label indications for coronary IVL.

**Table 5:** – Comparison of DISRUPT CAD I-IV patient-level pool analyses with the current study

Characteristics	Current Study (n=104)	DISRUPT CAD I-IV (n = 628)
**Age (years)**	73.2 ±10.2	71.8 ± 8.9
**Male**	79 (76)	484 (77.1)
**Diabetes mellitus**	46 (44.2)	241 (38.4)
**Hypertension**	82 (78.8)	539 (85.8)
**Prior MI**	23 (22.1)	137 (21.8)
**Prior coronary artery bypass grafting**	14 (13.5)	60 (9.6)
**Severe renal insufficiency (eGFR<30 ml/min/1.73 m2)**	6 (5.8)	Excluded
**Acute coronary syndromes**	65 (62.5)	Excluded
**Intravascular imaging, %**	78	54
**Lesion length (mm)**	21.7 ± 11.7	24.4 ± 11.5
**Side branch involvement**	65 (51.1)	190 (30.3)
**Angiographic severe coronary calcification**	93 (73.2)	609 (97.0)
**Unprotected left main PCI assisted by IVL**	9 (7.1)	Excluded
**Final in-stent angiographic outcomes**		
Acute gain, mm	1.55 ± 0.79	1.68 ± 0.47
Minimum lumen diameter, mm	3.06 ± 1.79	2.75 ± 0.44
Residual stenosis < 30%	96.8	95.7 (97.7–99.6)
**Major adverse cardiac events, %**	4.8	6.5 (4.7–8.8)
Severe angiographic complications,* %	1.9	2.1

Numbers are counts (percentages) or means (± standard deviations). eGFR: estimated glomerular filtration rate; IVL: intravascular lithotripsy; MI: myocardial infarction; PCI: percutaneous coronary intervention. * Dissections ≥ grade D, perforations, abrupt closure, and slow flow/no-reflow.

The present study has some limitations that should be underscored. This is a prospective single-center non-randomized study; therefore, we cannot exclude any biased selection, and the favorable outcomes found here must be interpreted with caution. However, the results add to the growing body of worldwide data on coronary lithotripsy being used in different populations, which is essential for every recent technology. Combined methods with IVL, such as other ablative devices, have been shown to be safe in this small series of cases, but this requires confirmation in larger controlled clinical trials as an efficient approach. All procedures were performed by operators experienced in complex PCI, with high utilization of intravascular imaging, which may have contributed to the favorable outcomes regardless of IVL therapy. However, given the absence of systematically collected intravascular imaging-derived quantitative endpoints and the lack of a control group for comparison, any association with the favorable outcomes observed remains hypothesis-generating. Notably, this was the first time all operators used coronary lithotripsy, which proved to be a practical tool, although long-term follow-up is essential to evaluate the persistence of the observed in-hospital efficacy. Finally, comparison between our population and other registries can only be speculative.

## Conclusions

This study presents the initial experience with PCI assisted by IVL for the treatment of severely calcified coronary lesions at a high-volume cardiovascular center. Over time, we observed a reduction in angiographic complications, particularly dissections, between the early and late cohorts, supporting a short learning-adaptation curve and increasing operator familiarity with the technique. The later cohort also required a longer enrolment period to achieve a similar sample size, which may reflect progressively more selective IVL utilization as institutional experience matured. Importantly, in-hospital adverse clinical events were infrequent and did not differ between temporal cohorts. Taken together, these findings support the safety and procedural effectiveness of IVL-assisted PCI in routine practice during the early adoption phase, while highlighting the relevance of operator experience and case selection.
